# Balloon-Occluded Retrograde Transvenous Obliteration (BRTO) for Gastric Varices: A Single-Center Experience in the Middle East

**DOI:** 10.7759/cureus.83726

**Published:** 2025-05-08

**Authors:** Muneera Almohannadi, Ahmed Omar, Ali Barah, Anwar I Joudeh, Ahmed M Badi, Yousra Ali, Yousef Yahia, Rajvir Singh, Betsy Varughese, Rafie Yakoob

**Affiliations:** 1 Gastroenterology and Hepatology, Hamad Medical Corporation, Doha, QAT; 2 Radiology, Hamad Medical Corporation, Doha, QAT; 3 Internal Medicine, Hamad Medical Corporation, Doha, QAT; 4 Acute Care Surgery, Hamad Medical Corporation, Doha, QAT

**Keywords:** brto, embolization, gastric varices, liver cirrhosis, portal hypertension, portosystemic shunt, variceal bleeding

## Abstract

Aims: This study aims to assess the short- and long-term outcomes of balloon-occluded retrograde transvenous obliteration (BRTO) in managing gastric varices (GV).

Methods: We conducted a retrospective chart review of all patients who underwent BRTO for actively bleeding or at high-hemorrhagic-risk GV secondary to liver cirrhosis at our facility from January 2007 to December 2019. Descriptive and analytical statistics were used to evaluate short- and long-term postprocedural outcomes. Univariate and multivariate analyses were used to identify significant factors associated with mortality. Survival was assessed using the Kaplan-Meier method. A p-value of <0.05 was considered statistically significant.

Results: A total of 35 patients (mean age: 53.3 ± 9.9 years) were included, with the majority being men (N = 29, 82.9%). Most patients had Child-Pugh classification A (N = 14, 40%) or B (N = 13, 37.1%). Twenty-two patients (62.9%) underwent BRTO emergently, and 13 (37.1%) underwent the procedure prophylactically. Collateral embolization and hemostasis were achieved in all but one patient. Gastric variceal recurrence occurred in five patients (14%), and esophageal varices (EV) worsened in three (9%). During a mean follow-up of 96.2 ± 9 months, seven patients (20%) experienced variceal bleeding episodes, all managed endoscopically. The estimated post-BRTO survival rates at 1, 3, 5, and 10 years were 82.1%, 82.1%, 76.6%, and 68.1%, respectively. Preprocedural Child-Pugh classification A or B and total bilirubin levels < 3.5 mg/dL were associated with better survival rates.

Conclusion: BRTO is a safe and effective treatment for both emergent bleeding control and prophylactic management of high-hemorrhagic-risk GV.

## Introduction

Gastric varices (GVs) are abnormally dilated submucosal veins that occur secondary to complex patterns of portosplenic and systemic venous shunts. GVs can be observed in portal hypertension due to liver cirrhosis, portal vein occlusion, or splenic vein thrombosis. The anatomical location and association with esophageal varices (EVs) vary according to the underlying etiology [1]. Sarin classifications endoscopically categorize GVs into four types according to their location: gastroesophageal varices type 1 (GOV-1), extending from the esophagus to the lesser curvature; gastroesophageal varices type 2 (GOV-2), extending from the esophagus to the fundus; isolated gastric varices type 1 (IGV-1), involving the gastric fundus; and isolated gastric varices type 2 (IGV-2), involving other parts of the stomach. The most frequent type of GV in liver cirrhosis is GOV-1, whereas GOV-2 and IGV-1 are more common in portal or splenic vein thrombosis [2]. 

Although the prevalence of GVs is much lower than that of EVs, they are more prone to bleeding and more challenging to treat [3,4]. GVs account for only one-fifth of variceal bleeding cases, but the mortality rate is higher, ranging from 25% to 55% [5]. Due to the heterogeneity of the underlying shunts, treatment approaches and prognosis are variable. GVs drain via azygos/hemiazygos veins to the superior vena cava (GOV1), or through gastrorenal (GRS) and gastrocaval shunts into the left renal vein or inferior vena cava (mainly GOV2 and IGV1); IGV2 drains back into the portal system [4]. Several surgical, endoscopic, and radiological interventions are utilized, including shunt surgery, splenectomy, injection sclerotherapy, and transjugular intrahepatic portosystemic shunts (TIPS). However, each of these modalities has limitations in safety, efficacy, or applicability based on the clinical context [6]. Invasive shunt surgery is contraindicated for many patients with poor hepatic functional reserve [7]. The rapid intra-variceal blood flow causes a rapid loss of the sclerosing agents in endoscopic injection sclerotherapy [8]. The application of TIPS is also not successful in all patients as it does not result in complete regression of the GV and might lead to worsening of hepatic encephalopathy [9].

Balloon-occluded retrograde transvenous obliteration (BRTO) was first developed in Japan in the mid-1990s as a minimally invasive radiologic technique for managing GV, particularly in patients with large GRS shunts [10]. It gained widespread use in Korea shortly thereafter and was introduced in the United States in the early 2000s. Subsequent procedural refinements in 2013 and 2014, including improved catheter systems and adjunctive embolization techniques, helped simplify the approach and reduce complications. BRTO works by occluding portosystemic shunts, thereby redirecting blood flow into the portal system, which can improve hepatic perfusion and liver function. Early observational studies demonstrated high technical success and effective bleeding control. However, concerns were raised about complications such as worsening of EVs, portal hypertension, and rare cases of systemic venous thrombosis [10-19]. Several studies have specifically reported that BRTO may lead to progression or exacerbation of EVs due to the increase in portal venous pressure and the redistribution of blood flow following shunt occlusion, highlighting the importance of close postprocedural endoscopic surveillance and appropriate prophylactic interventions [11-13,15-17]. Despite these concerns, BRTO has increasingly been recognized in international guidelines. According to the 2024 American Association for the Study of Liver Diseases (AASLD) guidelines on managing gastric variceal hemorrhage, cyanoacrylate injection, TIPS, or BRTO are considered first-line options. BRTO is particularly favored in patients with contraindications to TIPS, such as those at high risk of hepatic encephalopathy or with poor hepatic reserve, while TIPS remains preferred in patients requiring broader decompression of portal hypertension.

Revised objectives

The primary objective of this study is to evaluate the safety and effectiveness of BRTO in the management of GVs. Specifically, we aim to assess key clinical outcomes, including mortality, rebleeding rates, GV recurrence, and complications such as worsening of EVs. In addition, the study examines long-term survival following BRTO and compares these outcomes to those reported in prior literature. By providing region-specific data, this study also seeks to inform clinical decision-making in areas where evidence on BRTO remains limited, particularly in the Middle East.

This article was previously posted on the medRxiv preprint server on April 27, 2020 (available at https://www.researchsquare.com/article/rs-3404300/v1).

## Materials and methods

Study design and population

This retrospective observational study included all adult patients with GV referred to the Department of Interventional Radiology at Hamad Medical Corporation, Doha, Qatar, from January 2007 to December 2019. Hamad Medical Corporation is Qatar's main secondary and tertiary care provider, including 12 hospitals throughout the country. We did not calculate the sample size as we included all patients who underwent the procedure in our facility, except for one with only ectopic varices in the small bowel.

**Figure 1:**
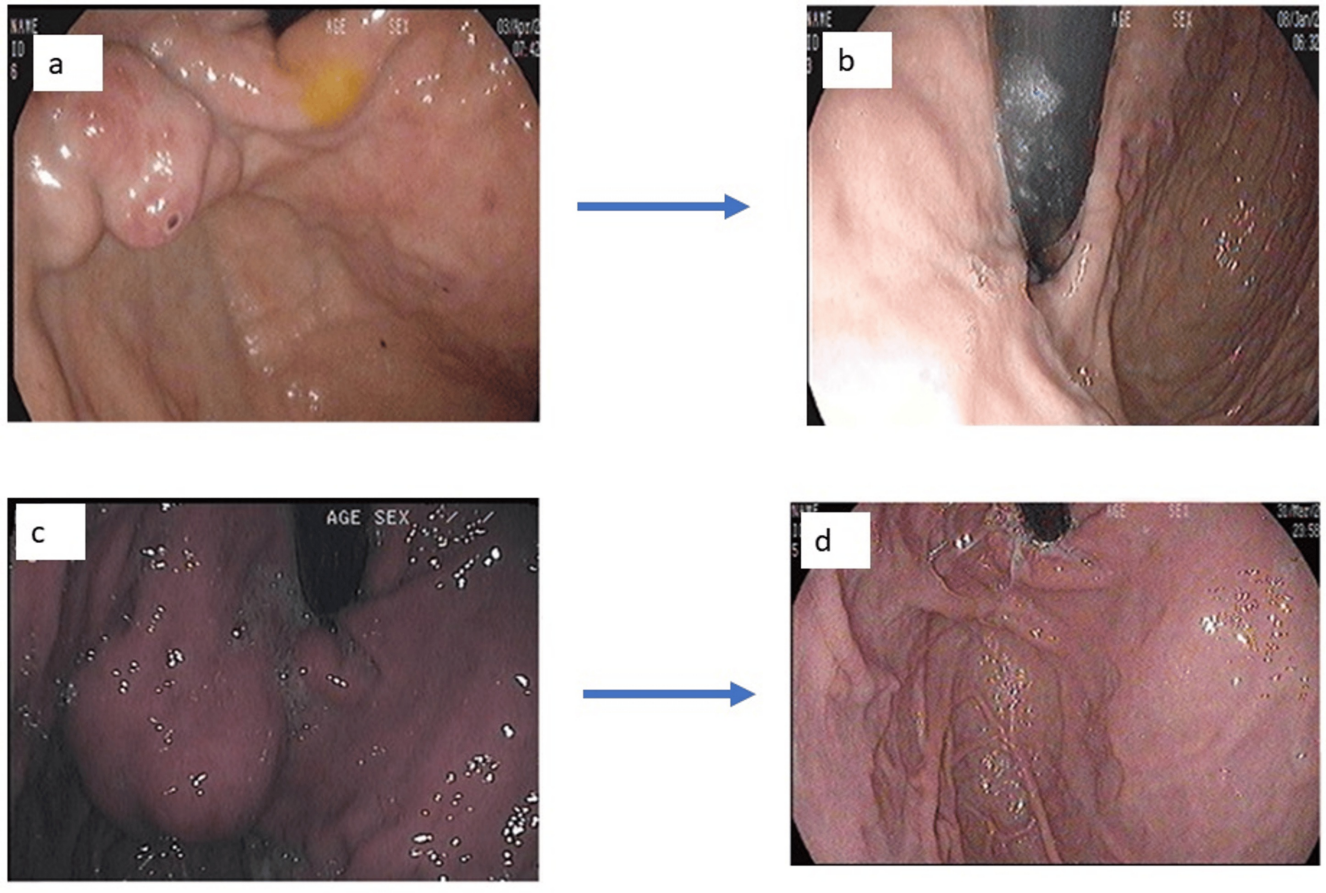
Endoscopic view of fundal varices before and after balloon-occluded retrograde transvenous obliteration (BRTO) procedure (a, b) The patient underwent an emergency BRTO procedure for active bleeding from gastric varices. (c, d) The patient underwent a prophylactic BRTO procedure for large gastric varices.

Patients who were referred for BRTO had either active GV bleeding that was initially stabilized by endoscopic glue injection and underwent BRTO within 24 hours of the bleeding episode or were referred electively for BRTO after diagnosing high-risk GV on surveillance endoscopy (Figure 1). The GRS shunt was confirmed by cross-sectional imaging before the procedure. Contraindications to BRTO included portal vein thrombosis, severe hepatic dysfunction, severe coagulopathy, and severe renal failure. All patients who underwent BRTO treatment were followed throughout the study period. Laboratory investigations, including full blood count, coagulation profile, and renal and liver function tests, were done within 24 hours of the BRTO procedure and were repeated after 30 days.

Esophagogastroduodenoscopy (OGD) was repeated urgently in case of clinical suspicion for upper gastrointestinal bleeding and periodically according to the individual patient’s risk and endoscopic findings. OGD was repeated routinely after 4-6 weeks to confirm the eradication of GV. Patients with baseline EV before BRTO underwent endoscopy within two weeks for high-risk EVs and within 4-6 weeks for low-risk EVs. Worsening EVs were defined as having a new-onset EV or the presence of red spots or bleeding from a previously diagnosed EV on follow-up endoscopic evaluation. The duration of the follow-up period was decided by the attending gastroenterologist and according to the individual patient’s risk factors for bleeding.

The follow-up regimen included monitoring of recurrence of GV, worsening of EV, variceal bleeding, and survival rate for the study duration.

Data acquisition

We conducted a comprehensive chart review of electronic medical records for all included patients. Data extraction was carried out using a combination of structured electronic queries and manual chart review to ensure completeness. A standardized data collection form was developed and used throughout the process to maintain consistency across records. Extracted variables included demographic characteristics, medical comorbidities, underlying etiology of liver cirrhosis, Child-Pugh classification, liver failure-related complications (hepatic encephalopathy, ascites, spontaneous bacterial peritonitis (SBP), and hepatocellular carcinoma (HCC)), endoscopic classification of GV, BRTO procedure type (emergency vs. prophylactic), vascular access site, and laboratory parameters (complete blood count, renal function, liver function, and coagulation profile).

To enhance data reliability and minimize observer bias, two independent reviewers cross-validated a random subset of records. Any discrepancies were resolved through consensus or consultation with a third senior investigator. Data on short-term complications (≤30 days), such as technical difficulties, renal dysfunction, and pulmonary edema, and long-term complications (>30 days), including variceal rebleeding, recurrence of GV, worsening of EVs, and all-cause mortality, were systematically collected and recorded.

The BRTO procedure was defined as an emergency procedure if it was performed in patients with at least one bleeding episode from GV confirmed by endoscopic examination. Prophylactic BRTO was defined as having the procedure performed in the absence of a previous bleeding episode and in the presence of an endoscopically documented high-risk bleeding GV according to the GV size, site, and the presence of high-risk stigmata such as discolored marks and platelet plugs [4]. While coil embolization of additional collateral veins was performed based on patient-specific anatomy, this variation was not anticipated to affect the primary outcomes and was not stratified for subgroup analysis. All BRTO procedures were performed under fluoroscopic guidance following a triple-phase contrast-enhanced CT for vascular mapping. The sclerosant used was ethanolamine oleate 5% mixed with contrast (10-15 cc), and the balloon was kept inflated for approximately four hours post-sclerosant injection. Stasis was confirmed via venogram before balloon deflation.

BRTO procedure

A written informed consent for the BRTO procedure was obtained from all patients or their legal guardians. Before the procedure, all patients underwent a triple-phase enhanced CT to identify the variceal efferent and afferent veins and map the vascular anatomy of the GRS shunt, other portosystemic shunts, and the portal vein anatomy. All BRTO procedures were performed under conscious sedation using intravenous fentanyl and midazolam. The procedure was standardized between all patients, with the only variation being the use of coil embolization of different variceal collaterals (e.g., pericardial, paravertebral, or phrenic veins) according to the patient’s variceal anatomic variants.

**Figure 2:**
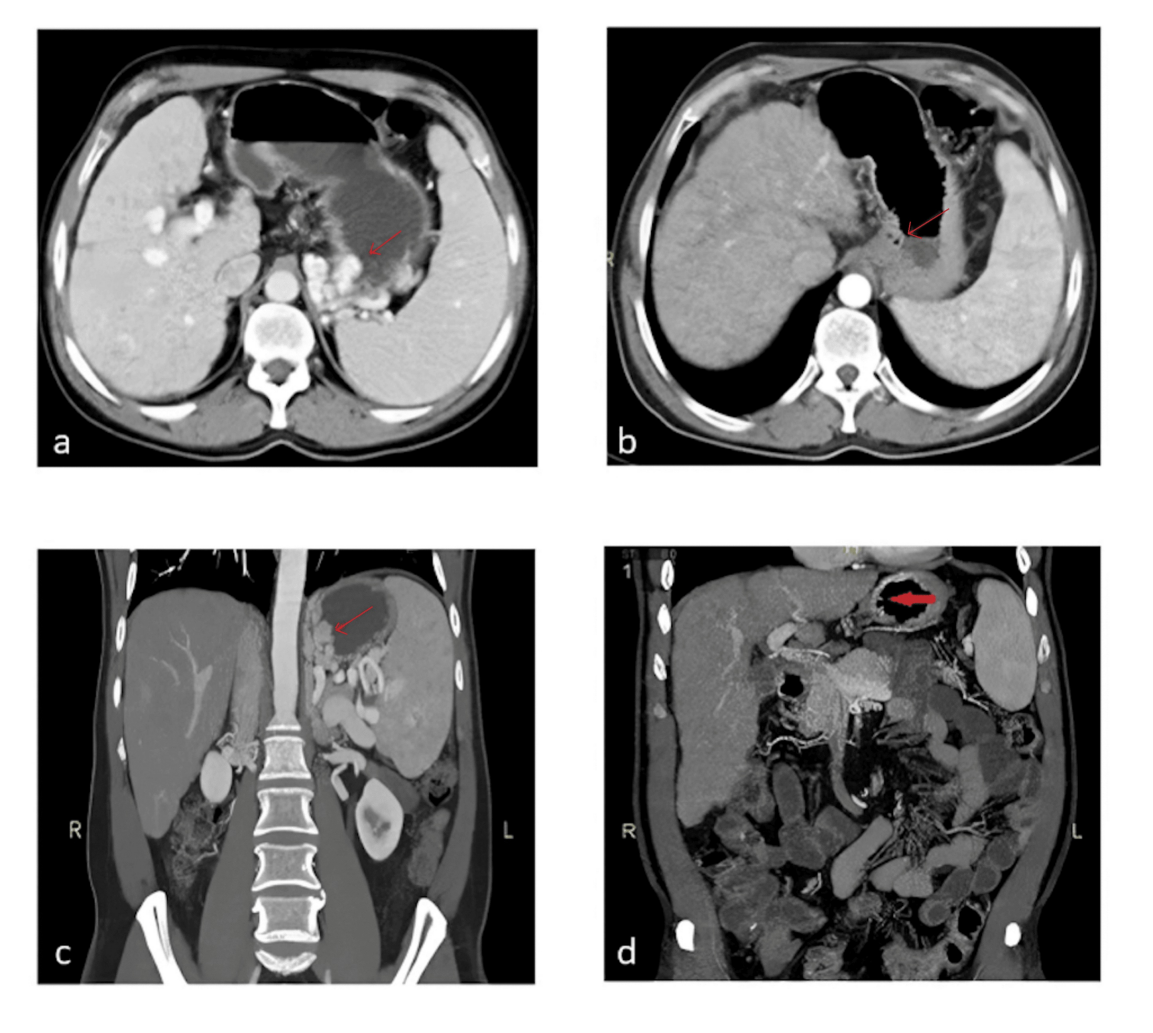
Contrast-enhanced computed tomography (CT) scan images of patients before and after balloon-occluded retrograde transvenous obliteration (BRTO) procedure (a) Contrast-enhanced CT (CECT) scan showing gastric fundal varices (arrow). (b) Post-BRTO CECT axial image showing total obliteration of the gastric fundal varices (arrow). (c) Coronal reconstruction of the CECT scan showing the gastric fundal varices (arrow). (d) Post-BRTO procedure CECT coronal image showing total obliteration of the gastric fundal varices.

**Figure 3:**
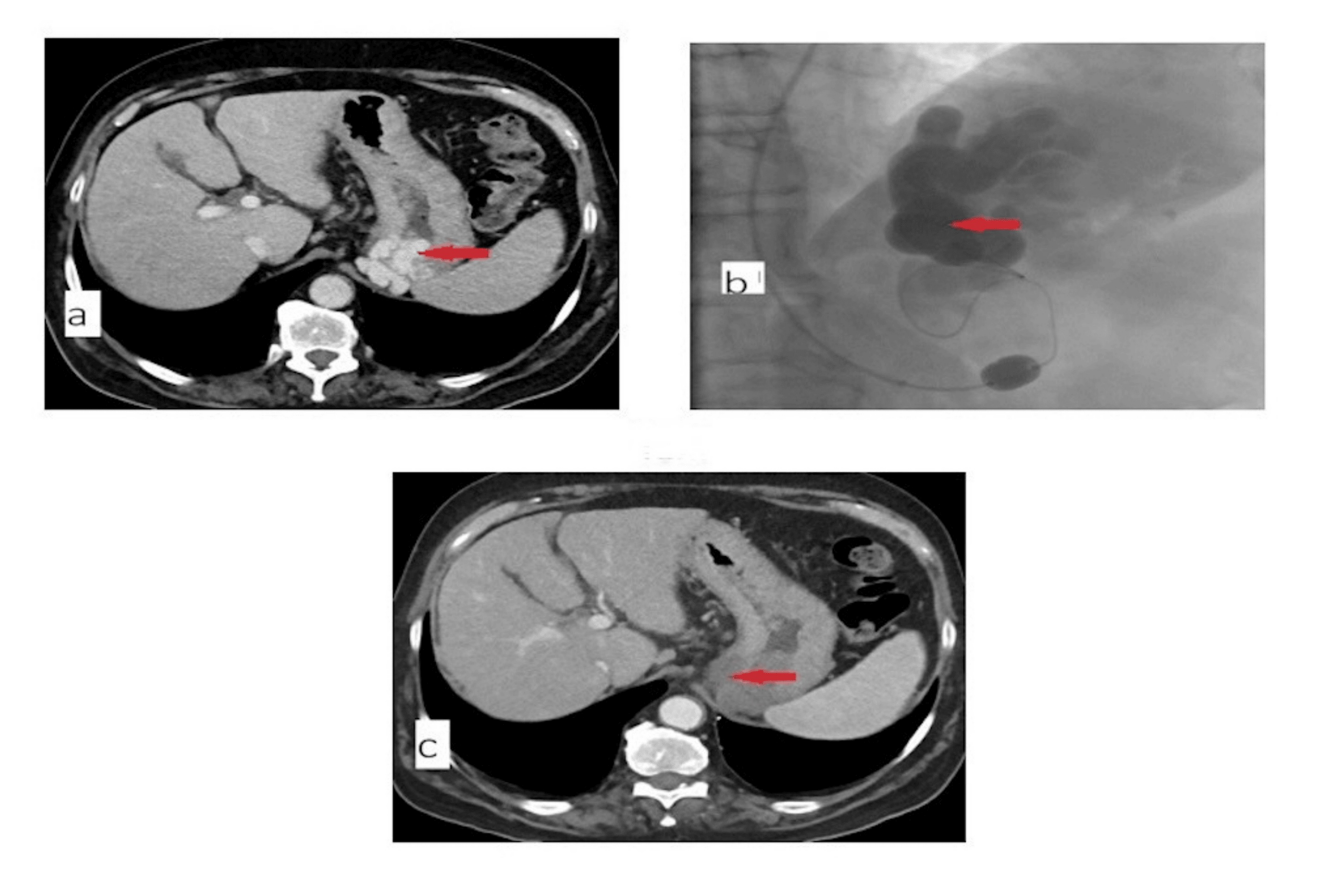
Contrast-enhanced computed tomography (CT) scan images of patients before and after balloon-occluded retrograde transvenous obliteration (BRTO) procedure (a) Preprocedural contrast-enhanced CT showing gastric fundal varices (arrow). (b) Fluoroscopy image during BRTO showing contrast filling into the gastric varices and gastrorenal shunt (image created by the authors for educational purposes). All study procedures were performed via the femoral approach. (c) Postprocedural contrast-enhanced CT showing total obliteration of the gastric fundal varices (arrow).

In 34 out of 35 patients, the right femoral vein was accessed with the insertion of a 5-Fr vascular sheath, and in one patient, the left femoral vein was accessed due to technical difficulties. The right renal vein was engaged using a 5-Fr Cobra 2 catheter (AngioDynamics, Latham, NY, United States) over a hydrophilic-coated 0.035-inch guidewire (Terumo, Tokyo), followed by engagement of the GRS shunt using the same catheter. Alternatively, a 5-F Vertebral catheter (Terumo, Tokyo) or 5F Siemens II (AngioDynamics, Latham, NY, United States) was used in case of failure with a Cobra catheter. Next, the hydrophilic guidewire was advanced into the GRS shunt, followed by the catheter advancement into the varices. Contrast is then injected to confirm the position within the shunt. The hydrophilic guidewire was then exchanged with a stiff Amplatz 0.035-inch guidewire (Boston Scientific, Marlborough, MA, United States), followed by retrieval of the Cobra catheter. The femoral 5F vascular sheath was then replaced by a 14F introducer sheath. A 7F-compliant Equalizer occlusion balloon catheter (Boston Scientific, Marlborough, MA, United States) was then advanced into the GRS shunt. The diameter of the balloon was selected based on the size of the shunt to be occluded. The balloon was then gently inflated and pulled back to make sure it was placed in the appropriate position, concluding the shunt. A balloon-occluded retrograde venogram of the varices was then performed to further evaluate the variceal anatomy and collaterals and to assess the need for coil embolization of any existing collateral using a microcatheter. In the presence of large-sized leaking collateral veins, selective catheterization using a microcatheter and embolization using metallic coils (Nester or Tornado coils; Cook Medical, Bloomington, IN, United States) were performed to occlude the collaterals. Finally, a sclerosant agent is injected through the microcatheter after it is advanced as far as possible within the varices. Our sclerosing agent was ethanolamine oleate 5% mixed with contrast, given in amounts ranging from 10 to 15 cc. The embolization endpoint was the minimal opacification of afferent veins. After embolization, the patient was sent to the recovery area for four hours with the balloon inflated. The patient was then brought back to the angio-suite, and a venogram was done to confirm contrast stasis within the varix. Once the stasis of contrast was achieved, the procedure was concluded. If no stasis was completed, more sclerosant agent was injected until satisfactory embolization of the gastric varices. A CT venogram was done the next day to further assess the outcome of the embolization, and clinical follow-up was done in all patients to assess the long-term outcome of the BRTO procedure (Figure 2, Figure 3). 

Statistical analysis

Descriptive statistics were calculated using means, standard deviations, and interquartile ranges (IQRs) for continuous variables and frequencies with percentages for categorical variables. Paired comparisons of pre- and postprocedural continuous variables were conducted using Student's t-tests or Wilcoxon signed-rank tests, as appropriate. Between-group comparisons (e.g., mortality vs. no mortality) were performed using unpaired t-tests for continuous variables (e.g., age, bilirubin, INR) and chi-square tests for categorical variables (e.g., gender, etiology of cirrhosis). A multivariate logistic regression model was employed to identify independent risk factors associated with mortality.

Follow-up duration ranged from 1 to 154 months, and all patient time-to-event data were appropriately censored at the time of last known follow-up. Kaplan-Meier survival analyses were conducted to estimate median survival for mortality and recurrence-free survival for gastric varices. A two-tailed p-value of <0.05 was considered statistically significant. All statistical analyses were performed using IBM SPSS Statistics for Windows, Version 29.0 (Released 2023; IBM Corp., Armonk, NY, United States).

## Results

As shown in Table 1, 29 out of the 35 patients included in the study were men (82.9%), with a mean age of 53.3 ± 9.9 years. The most common etiologies for underlying liver cirrhosis were hepatitis C and cryptogenic cirrhosis (42.9% and 31.4%, respectively). At baseline, most of the cases had a Child-Pugh classification A or B (40% and 37.1%, respectively), four patients had liver transplants (11.4%), and one patient had hepatocellular carcinoma (2.9%). Two-thirds of the patients had emergency BRTO procedures for bleeding GV. Regarding the type of GV, two-thirds of the patients had gastric fundal and EVs (60%), whereas the rest had isolated gastric fundal varices (40%).

**Table 1:** Demographic and clinical characteristics of the study participants at the time of balloon-occluded retrograde transvenous obliteration (BRTO) procedure IGV1: Isolated gastric fundal varices, GOV2: gastroesophageal varices extending into the gastric fundus.

Variable	Category	Number (%) or mean ± SD
Gender	Male	29 (82.9)
	Female	6 (17.1)
Age		53.3 ± 9.9 years
Underlying etiology for liver cirrhosis	Hepatitis C	15 (42.9)
	Hepatitis B	3 (8.6)
	Alcoholic liver disease	6 (17.1)
	Cryptogenic cirrhosis	11 (31.4)
Child-Pugh classification	A	14 (40)
	B	13 (37.1)
	C	8 (22.9)
Comorbidities	Diabetes mellitus	21 (60)
	Hypertension	12 (34.3)
	Coronary artery disease	3 (8.6)
Hepatic failure-related complications	Ascites	10 (28.6)
	Hepatic encephalopathy	8 (22.9)
	Spontaneous bacterial peritonitis	2 (5.7)
	Hepatocellular carcinoma	1 (2.9)
	Liver transplant	4 (11.4)
Type of gastric varices	Isolated gastric fundal varices (IGV-1)	14 (40)
	Gastric fundal varices and esophageal varices (GOV-2)	21 (60)
Type of procedure	Emergency for bleeding	22 (62.9)
	Prophylaxis	13 (37.1)

The mean follow-up period for this study was 96.2 ± 9 months. Table 2 shows the short (≤30 days)- and long (>30 days)-term complications that were observed during the study period. Within the first month post-BRTO, a follow-up CT showed embolization of the collateral channels and immediate hemostasis in all but one patient. Another patient had a recurrence of GV and bleeding within one month, and one patient had to have a repeated procedure due to technical difficulties. One patient died within one month of BRTO due to multiorgan failure, including renal dysfunction. He was 34 years old and had type 2 diabetes mellitus and liver cirrhosis secondary to a hepatitis B infection. However, we did not observe any other case of acute kidney injury or other potential immediate complications such as fever, anaphylactic reactions, pulmonary edema, hemothorax, or hydrothorax.

Regarding long-term complications, four patients had a recurrence of GVs, two had a worsening of EVS, and one had both a recurrence of GV and a worsening of EVs. Variceal bleeding occurred in six patients, including four who had previous episodes of GVs and two who had the procedure prophylactically. All bleeding episodes were successfully treated endoscopically. Although eight patients (22.9%) had encephalopathy and 10 patients (28.6%) had ascites at initial admission, neither condition was exacerbated after the procedure. Two patients developed hepatocellular carcinoma during follow-up (at 14 and 18 months post-procedure, respectively); these cases were deemed unrelated to BRTO and were considered expected given the underlying cirrhosis. Another six patients died during the long-term follow-up period: one patient died due to cardiac arrest, two patients due to hepatocellular carcinoma progression, two patients due to advanced age and progression of their multiple comorbidities, and one patient due to liver transplant rejection because of medication noncompliance.

**Table 2:** Short- and long-term complications of balloon-occluded retrograde transvenous obliteration (BRTO) procedure *Technical complications are defined as the need to repeat the procedure. **Rebleeding frequency was counted based on the denominator of 22 cases (number of patients who had emergency BRTO procedure for bleeding gastric varices).

Category	Short-term (≤ 30 days)	Long-term (>30 days)	Total number (percentage %)
Anaphylaxis	0	0	0 (0)
Renal dysfunction	1	0	1 (2.9)
Disseminated intravascular coagulation	1	0	1 (2.9)
Technical complications*	1	0	1 (2.9)
Gastric varices recurrence	1	4	5 (14)
Worsening of esophageal varices	0	3	3 (9)
Rebleeding**	1	4	5 (23)
Total variceal bleeding	1	6	7 (20)
All-cause mortality	1	6	7 (20)

Table 3 demonstrates selected laboratory parameters immediately before and one month after the BRTO procedure. Albumin levels increased significantly after the BRTO procedure (mean ± SD: 34.4 ± 7.4 gm/L vs. 31.3± 6.7 gm/L, p = 0.004), but there was no significant change in their bilirubin levels (median (range): 1.37 (0.82-3) mg/dL vs. 1.11 (0.64-2.34) mg/dL, p = 0.22), prothrombin time (PT) (mean ± SD: 12.9 ± 4.0 seconds vs. 13.6 ± 2.8 seconds, p = 0.10), and creatinine levels (median (range): 66 (54-80.50) umol/L vs. 64.5 (54.5-73.25) umol/L, p= 0.26). International normalized ratio (INR) was statistically significantly reduced after the procedure (mean ± SD: 1.29 ± 0.42 vs. 1.34 ± 0.24, p = 0.01). However, these biochemical parameters were within the normal clinical range in all patients after the procedure.

**Table 3:** Comparison between laboratory investigations before and after balloon-occluded retrograde transvenous obliteration (BRTO) procedure (N = 35) BRTO: balloon-occluded retrograde transvenous obliteration, PT: prothrombin time, INR: international normalized ratio. Laboratory investigations were done within 24 hours pre-BRTO procedures and 30 days following the procedure. The t-test (paired) or Wilcoxon signed-rank test was used to compare pre- and post-intervention results. *p < 0.05 is considered statistically significant.

Laboratory investigation	Pre-BRTO procedure	Post-BRTO procedure	t-value, degrees of freedom (df)	p-value
Bilirubin (umol/L) (median, IQR)	23.50 (14.00-51.25)	19 (11.0-40.0)	-1.28, 32	0.22
PT (seconds) (mean ± SD)	13.6 ± 2.8	12.9 ± 4.0	1.56, 32	0.10
INR (mean ± SD)	1.34 ± 0.24	1.29 ± 0.42	0.90, 32	0.01*
Creatinine (umol/L) (median, IQR)	64.5 (54.5-73.25)	66 (54.0-80.50)	-0.95, 32	0.26
Albumin (gm/L) (mean ± SD)	31.3 ± 6.7	34.4 ± 7.4	-3.50, 33	0.004*

Table 4 shows the bivariate analysis for the associated factors with mortality post-BRTO procedure. As shown in the table, advanced classes of Child-Pugh classification and higher bilirubin levels (≥3.5 mg/dL) were statistically significantly associated with higher mortality rates (p = 0.02 and p = 0.04, respectively). Factors such as age, gender, underlying cause for liver cirrhosis, or the type of procedure (emergency vs. prophylaxis) did not have statistically significant associations with mortality. However, multivariate analysis showed that only Child-Pugh classifications A and B were statistically and significantly associated with a lower risk of post-procedure mortality (OR: 0.09, 95% CI 0.01-0.66, p = 0.02) (Table 5).

**Table 4:** Multivariate analysis for associated factors for mortality post-balloon-occluded retrograde transvenous obliteration (BRTO) procedure (N = 35) Student's t-test (unpaired) and chi-square tests were used. *p < 0.05 is considered statistically significant.

Variable	Category		Mortality			
		Yes (N = 7; 20%)		No (N = 28; 80%)	t-value or chi-square value, degrees of freedom(df)	p-value
Age	--	57 ± 13.7		52.6 ± 8.8	-0.58, 33	0.30
Gender	Male	6 (85.7)		23 (82.1)	0.05, 1	0.82
	Female	1 (14.3)		5 (17.9)		
Cause of liver cirrhosis	Hepatitis C	1 (14.3)		14 (50.0)	5.40, 3	0.15
	Hepatitis B	1 (14.3)		2 (7.1)		
	Alcoholic liver disease	3 (42.9)		3 (10.7)		
	Cryptogenic	2 (28.6)		9 (32.1)		
Child-Pugh classification	A	0 (0)		14( 50.0)	8.1, 2	0.02*
	B	3 (42.9)		10( 35.7)		
	C	4 (57.1)		4 (14.3)		
Comorbidities	Diabetes mellitus	5 (71.4)		2 (28.6)	0.47, 1	0.49
	Hypertension	2 (28.6)		5 (71.4)	0.12, 1	0.72
	Coronary artery disease	1 (14.3)		6 (85.7)	0.37, 1	0.50
Liver-related parameters	Encephalopathy	2 (28.6)		5 (71.4)	0.16, 1	0.68
	Ascites	2 (28.6)		5 (71.4)	0.0, 1	0.67
	Spontaneous bacterial peritonitis	0 (0)		2 (100)	0.53, 1	0.47
	Bilirubin level:					
	<3.5 mg/dL	4 (57.1)		26 (92.9)	5.83, 1	0.04*
	≥ 3.5 mg/dL	3 (42.9)		2 (7.1)		
Type of procedure	Emergency	6 (85.7)		16 (57.1)	1.96, 1	0.22
	Prophylaxis	1 (14.3)		12 (42.9)		

**Table 5:** Bivariate analysis to identify the associated factors with mortality following balloon-occluded retrograde transvenous obliteration (BRTO) procedure Student's t-test (unpaired) and chi-square tests were used. *p < 0.05 is considered statistically significant.

Variable	Adjusted OR	Wald statistics, degree of freedom(df)	95% CI	p-value
Age in years	1.10	2.17, 1	0.97-1.26	0.14
Male gender	9.63	2.62, 1	0.62-149.5	0.11
Bilirubin ≤ 3.5 mg/dL	0.32	1.35, 1	0.05-2.18	0.25
Child-Pugh A and B	0.09	5.57, 1	0.01-0.66	0.02*

**Figure 4:**
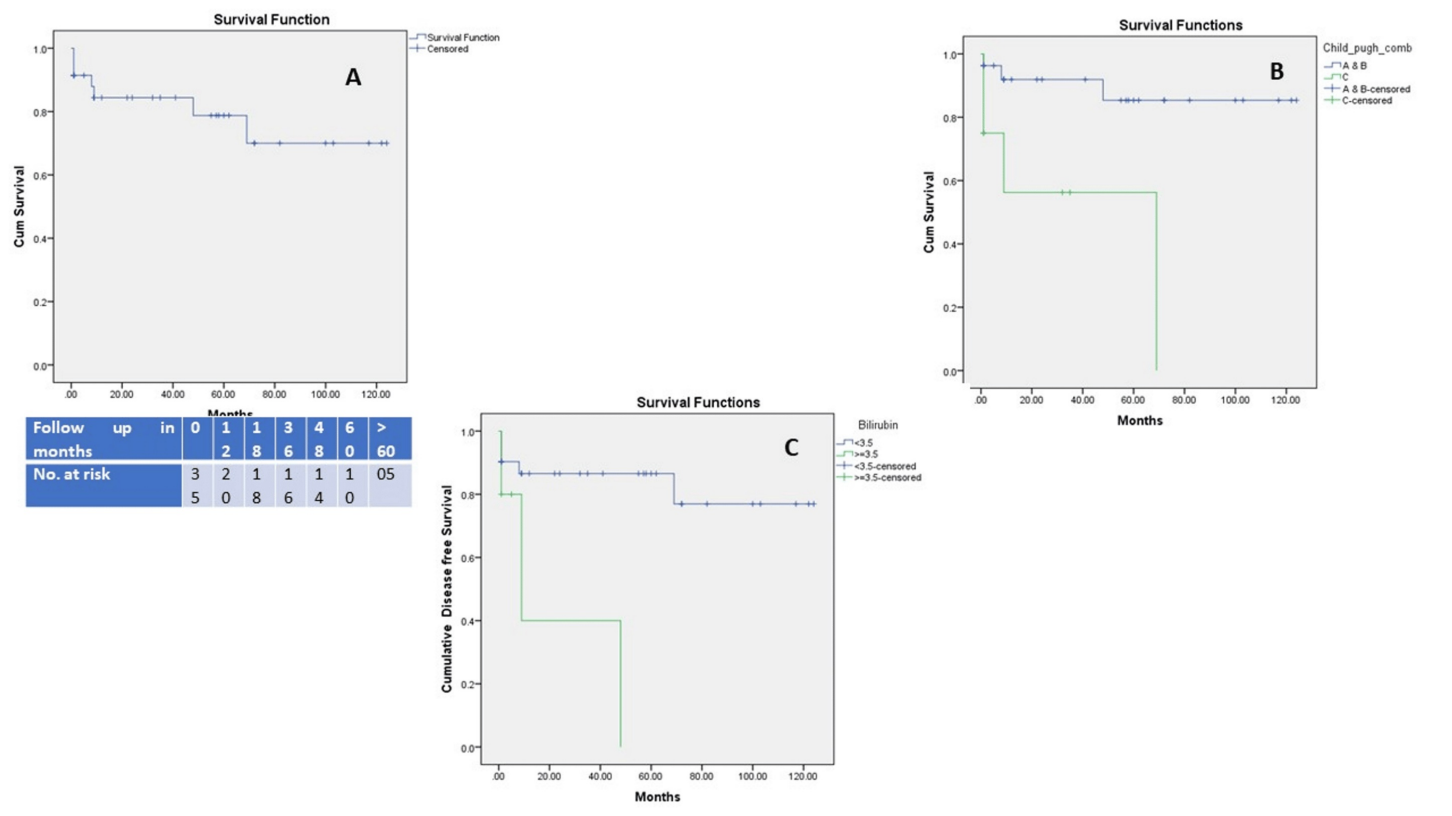
Kaplan-Meier survival curve showing survival rates after balloon-occluded retrograde transvenous obliteration (BRTO) (A) Cumulative survival rate of all patients after BRTO. The overall estimated mean follow-up was 96.2 ± 9.2 months, whereas the median survival was >124 months. B Survival rates according to preprocedural Child-Pugh classifications A and B (blue line) versus classification C (green line). The estimated mean survival time for Child-Pugh classification A and B is 109 ± 8 months, whereas the estimated survival rate for class C is 41 ± 15 months (p = 0.003). C Survival rates according to the preprocedural total bilirubin < 3.5 mg/dL (blue line) versus ≥ 3.5 mg/dL (green line). The median survival rate was more than 69 months in patients with bilirubin level < 3.5 mg/dL, whereas it was only 9 months in patients with bilirubin > 3.5 mg/dL (log-rank (Mantel-Cox), χ² = 7.66, p = 0.006).

**Figure 5:**
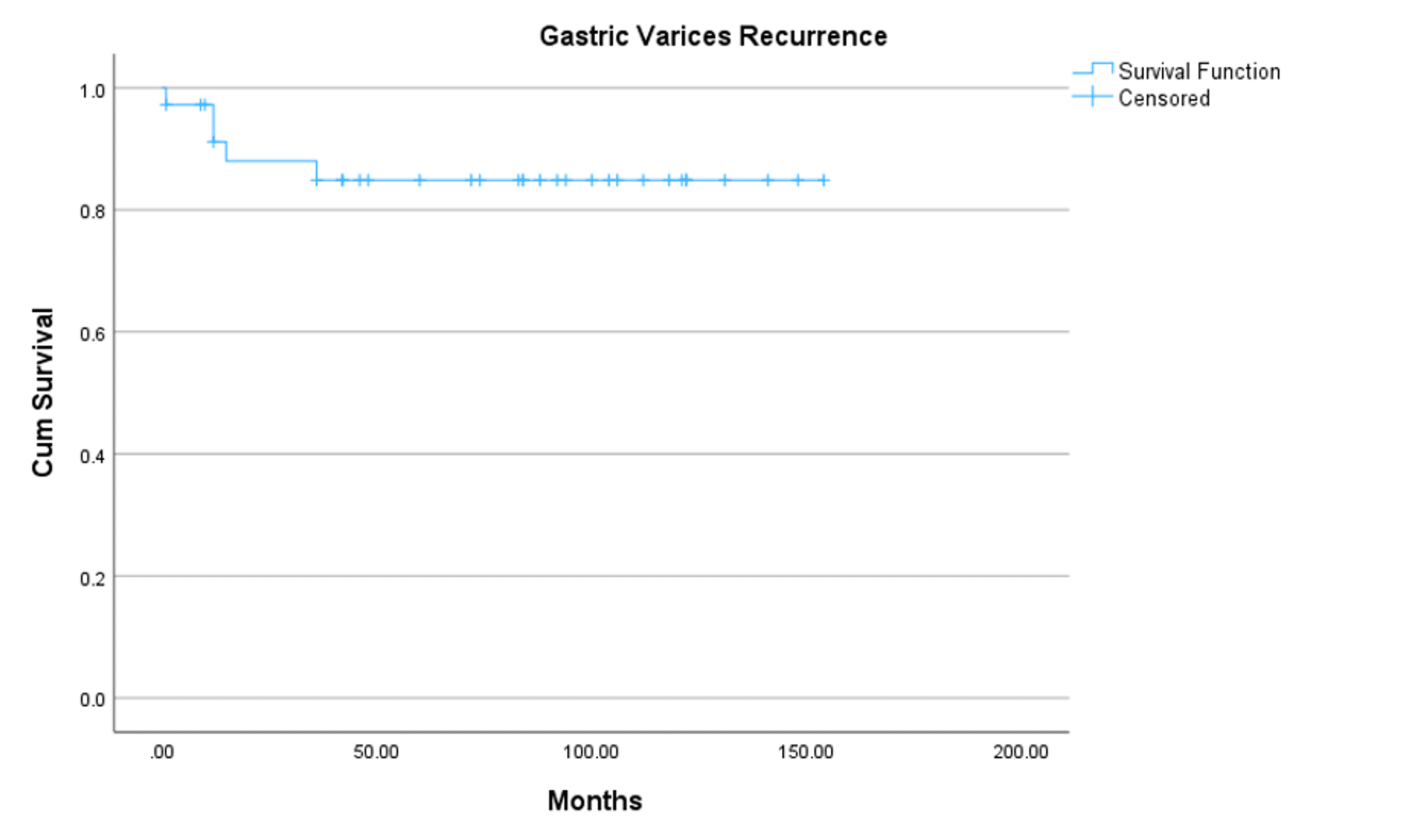
Kaplan-Meier curve demonstrating gastric varices recurrence-free survival following balloon-occluded retrograde transvenous obliteration (BRTO) The curve indicates the cumulative proportion of patients without variceal recurrence over time. Censored observations are marked with a “+” symbol. The mean recurrence-free survival time was 133 ± 8 months.

According to the Kaplan-Meier survival curve (Figure 4), the overall survival rates of the patients after the BRTO procedure were 82.1% at one year, 82.1% at three years, 76.6% at five years, and 68.1% at 10 years, with a cumulative survival rate of more than 124 months. Preprocedural Child-Pugh classifications (A and B) and preprocedural total bilirubin levels < 3.5 mg/dL were significantly associated with longer survival rates compared to preprocedural Child-Pugh classification C or bilirubin levels higher than 3.5 mg/dL with statistically significant results (109 ± 8 months vs. 41 ± 15 months, p = 0.003 and >69 months vs. 9 months p = 0.006, respectively). The Kaplan-Meier analysis (Figure 5) showed that the mean gastric varices recurrence-free survival following BRTO was 133 ± 8 months. Most recurrences occurred within the first 24 months, after which the recurrence rate plateaued with long-term follow-up. The cumulative recurrence-free survival remained above 85% beyond 10 years.

## Discussion

In this cohort of patients with GV, the BRTO procedure was safe and effective in securing bleeding with a high postprocedural survival rate of more than 10 years. Around one-quarter of patients had recurrence of GV bleeding, mostly in the long-term follow-up period (more than one month), but none of the bleeding episodes led to death. Ascites and encephalopathy did not increase after the procedure, and there was a statistically significant improvement in serum albumin and INR levels postprocedure, suggesting a potential enhancement in hepatic synthetic function. Higher bilirubin levels (>3.5 mg/dL) were associated with higher mortality rate and bleeding risk, whereas Child-Pugh classification C was associated with higher mortality risk.

Despite the technical demands of BRTO, we found reassuringly low rates of procedure-related complications. None of the patients in this cohort had serious adverse events such as anaphylaxis, fever, systemic venous thrombosis, worsening or new-onset ascites, or pleural effusion. Also, technical failure was reported in only one case, who later died due to multiorgan failure that was not directly related to the procedure. In comparison, a retrospective cohort study by Cho et al. [16] on 49 patients with GV secondary to liver cirrhosis reported higher rates of procedure-related deaths (2 patients), procedural failure (8), fever (23), portal vein thrombosis (2), pulmonary embolism (2), and left renal vein thrombosis (1). In addition, new-onset or worsening of pre-existing ascites occurred in 20 patients (44%), and new-onset pleural effusion in 33 patients (71%). Similar to our study, the mean age was 57 years, with most of the patients having liver cirrhosis secondary to viral hepatitis and alcoholic liver disease, with a Child-Pugh classification of A or B. However, all the patients included in the latter study had bleeding GV, with around 40% of them having hepatocellular carcinoma at baseline [16], while two-thirds of the patients included in this study had bleeding GV, and only one patient had HCC at the time of the procedure. Given the small number of patients and the retrospective nature of data collection, it would be difficult to interpret the differences in the short-term outcomes between the two studies, which warrants future randomized controlled trials to better evaluate the association between patients’ characteristics and clinical outcomes.

Similar to previous studies, we observed a small but statistically significant improvement in the liver synthetic function at the short-term follow-up. The observed post-BRTO decrease in bilirubin may reflect improved hepatic perfusion and metabolic function due to redirection of portal flow toward the liver, as described in prior studies [20]. No signs of hemolysis or liver decompensation were noted in our cohort. An earlier retrospective analysis of 29 patients who underwent successful BRTO showed significant improvement in the INR, bilirubin, and albumin levels in the few months after the procedure, with a consequent improvement in the model for end-stage liver disease (MELD) score. However, a significant number of patients had worsening ascites with or without hydrothorax, which negatively impacted their Child-Pugh classification [20]. On the other hand, other studies showed significant improvements in serum albumin levels [21] and encephalopathy following the BRTO procedure [6,11,12,22,23]. Interestingly, a study by Kumamoto et al. [22] analyzed the long-term effects of large splenorenal shunts on liver function and survival, where patients were divided into three groups: patients with no GRS shunt (the control group), patients with untreated GRS shunt, and patients with GRS shunts treated with BRTO. The patients with untreated GRS shunts had progressive deterioration of hepatic function than those in the BRTO treatment group and the control group. Conversely, patients who underwent BRTO had a transient improvement in hepatic function for 6-12 months and then returned to baseline hepatic function for up to three years. These patients had a stable hepatic function similar to the patients of the control group, which suggests that BRTO had a protective long-term role in preserving hepatic function and protecting the liver from portosystemic shunt syndrome [22]. Based on these findings, patients with larger degrees of GRS shunts and/or encephalopathy might have favorable outcomes of BRTO compared to those with advanced baseline levels of ascites, hydrothorax, and EV.

In this study, the BRTO procedure successfully controlled active bleeding in most patients with a low future risk of GV bleeding. Similarly, a systematic review and meta-analysis of 1,016 patients with bleeding or high-risk GV from 24 studies showed more than a 97% success rate in controlling GV bleeding with a low complication rate and low rebleeding rate of less than 5%-7% at one year [23]. However, around one-third of the patients in the latter review had recurrent EV. Tanihata et al. [24] studied the hemodynamic effects of BRTO on the portal systemic pressure gradient and its consequences on EV aggravation. Although the BRTO procedure resulted in the eradication of GV in all the study participants, EV was aggravated in 58% following the procedure. Higher baseline portal systemic pressure gradient and pretreatment EV were associated with higher risks for worsening of EV [24]. In contrast, less than 10% of the patients in this study had worsening EV, and all of them were treated successfully endoscopically. The lower rate of EV worsening observed in our cohort compared to previous studies may be attributed to several factors. First, our protocol's routine and early post-procedure endoscopic surveillance may have enabled earlier detection and management of EV progression, potentially preventing significant clinical worsening. Second, differences in baseline portal hemodynamics, patient selection (e.g., preserved liver function), or concurrent therapies (such as beta-blockers) may have contributed to mitigating EV exacerbation after BRTO. Conversely, the higher rate of GV recurrence in our study might reflect our longer follow-up duration, stricter radiologic and endoscopic definitions of recurrence, or due to a limited use of adjunctive embolization of collateral veins during the BRTO procedure. Incomplete embolization of alternative drainage pathways or the presence of complex collateral anatomy may have also contributed to recanalization and reformation of GV in some patients. The potential worsening of EV after BRTO warrants close monitoring and early management of baseline EV. According to the American Gastroenterological Association (AGA) treatment guidelines, the upper endoscopy should be repeated post-BRTO within 14 days and 4-6 weeks in patients with a baseline high-risk and low-risk EV, respectively [4].

Notably, the BRTO procedure was associated with high survival rates at both short- and long-term follow-up periods. The one-year survival rate of over 80% was consistent with previous studies [15,16,21,25], while the five-year (77%) and 10-year (68%) survival rates in our cohort were higher than those reported by Ninoi et al. (54%) [15], Waguri et al. (67% and 44%) [21], and Imai et al. (72%) [25]. However, it is important to note that BRTO does not directly improve overall survival; rather, it provides excellent bleeding control and prolongs rebleeding-free intervals. Long-term survival is primarily determined by the underlying liver function and volume of viable liver parenchyma. In our cohort, favorable baseline characteristics, such as a low proportion of patients with Child-Pugh class C, elevated bilirubin, or hepatocellular carcinoma (only one case at baseline), may have contributed to the higher observed survival rates. This underscores the importance of liver function status in predicting post-BRTO outcomes. Therefore, while BRTO plays a critical role in managing variceal bleeding, long-term prognosis is governed by the natural course of chronic liver disease.

Although most of the patients who had BRTO in this study had bleeding GVs, around one-third underwent BRTO prophylactically for high-risk GVs. We did not find significant differences in the short- or long-term outcomes between emergency or prophylaxis procedures. In alliance with our findings, the meta-analysis by Park et al. concluded that the technical and clinical success rates alongside EV recurrence rate in studies that evaluated BRTO for non-bleeding but at-risk GV were similar to the pooled rates from all the studies. Nevertheless, the authors could not conduct separate analyses for BRTO outcomes in acutely bleeding versus high-risk GV because most studies reported the results for both types without stratification [23]. Due to a lack of validated predictive tools for assessing bleeding risk from GV, the use of prophylactic interventions to prevent GV bleeding is still controversial, and many practice guidelines do not support specific primary prophylaxis approaches for GV [4,26]. This observation underscores the need for future research to validate the clinical and endoscopic predictors of GV bleeding risks.

This study is one of the few to examine the long-term outcomes of BRTO in patients with gastric varices in a Middle Eastern setting. However, several limitations must be acknowledged. First, the small sample size may reduce the statistical power and increase the risk of a type II error, particularly in subgroup analyses such as comparisons between emergent and prophylactic BRTOs. Second, the retrospective design introduces potential for selection bias, observer bias, and limitations in data completeness, despite our comprehensive review of electronic medical records. Third, although we reported statistically significant improvements in some liver function parameters (e.g., albumin and INR), the clinical relevance of these changes remains uncertain and requires further prospective validation. Fourth, our study was conducted at a single tertiary care center and reflects specific institutional practices and expertise, which may limit generalizability. Nonetheless, our findings are broadly consistent with previously published international data. Lastly, while EV worsening post-BRTO was infrequent in our cohort, differences in baseline portal pressure, follow-up duration, and procedural technique compared to other studies may explain this variation and warrant further investigation in multicenter studies.

## Conclusions

BRTO is an effective and safe procedure for preventing and controlling bleeding from GV in the presence of GRS shunts and the availability of local expertise. The risk of post-BRTO exacerbation of EV warrants close endoscopic surveillance. Poor prognostic factors affecting survival rates post-BRTO are largely related to the advanced liver disease parameters that patients have at baseline. Nevertheless, given the limitations of other endoscopic and endovascular interventions, BRTO provides a plausible interventional approach to control and prevent GV bleeding in the vulnerable population of liver cirrhosis. Larger, prospective, and controlled studies are needed to elucidate the role of BRTO in patients with GV and to validate endoscopic and clinical predictors for at-risk GV.
